# Genomic and phylogenetic features of the *Picobirnaviridae* suggest microbial rather than animal hosts

**DOI:** 10.1093/ve/veae033

**Published:** 2024-04-22

**Authors:** Sabrina Sadiq, Edward C Holmes, Jackie E Mahar

**Affiliations:** Sydney Institute for Infectious Diseases, School of Medical Sciences, The University of Sydney, Sydney, NSW 2006, Australia; Sydney Institute for Infectious Diseases, School of Medical Sciences, The University of Sydney, Sydney, NSW 2006, Australia; Laboratory of Data Discovery for Health Limited, Hong Kong, SAR, China; Sydney Institute for Infectious Diseases, School of Medical Sciences, The University of Sydney, Sydney, NSW 2006, Australia

**Keywords:** *Picobirnaviridae*, phylogeny, cross-species transmission, bacteriophage, classification, taxonomy

## Abstract

The RNA virus family *Picobirnaviridae* has traditionally been associated with the gastrointestinal systems of terrestrial mammals and birds, with the majority of viruses detected in animal stool samples. Metatranscriptomic studies of vertebrates, invertebrates, microbial communities, and environmental samples have resulted in an enormous expansion of the genomic and phylogenetic diversity of this family. Yet picobirnaviruses remain poorly classified, with only one genus and three species formally ratified by the International Committee of Virus Taxonomy. Additionally, an inability to culture picobirnaviruses in a laboratory setting or isolate them in animal tissue samples, combined with the presence of bacterial genetic motifs in their genomes, suggests that these viruses may represent RNA bacteriophage rather than being associated with animal infection. Utilising a data set of 2,286 picobirnaviruses sourced from mammals, birds, reptiles, fish, invertebrates, microbial communities, and environmental samples, we identified seven consistent phylogenetic clusters likely representing *Picobirnavirus* genera that we tentatively name ‘Alpha-’, ‘Beta-’, ‘Gamma-’, ‘Delta-’, ‘Epsilon-’, ‘Zeta-’, and ‘Etapicobirnavirus’. A statistical analysis of topological congruence between virus–host phylogenies revealed more frequent cross-species transmission than any other RNA virus family. In addition, bacterial ribosomal binding site motifs were more enriched in *Picobirnavirus* genomes than in the two groups of established RNA bacteriophage—the *Leviviricetes* and *Cystoviridae*. Overall, our findings support the hypothesis that the *Picobirnaviridae* have bacterial hosts and provide a lower-level taxonomic classification for this highly diverse and ubiquitous family of RNA viruses.

## Introduction

The analysis of metatranscriptomic data, either sequenced *de novo* or mined from the Sequence Read Archive, has greatly enhanced our knowledge of the scale, diversity, and composition of the RNA virosphere. As well as documenting huge numbers of novel viruses, this research programme has provided new insights into previously known families of RNA viruses. For example, major changes to taxonomic organisations have been proposed based on updated phylogenetic trees of RNA viruses (Shi et al. 2016; Wolf et al. 2020; Sadiq et al. 2022), and host ranges have been extended when viruses are detected in organisms with which they are not typically associated (Urayama, Takaki, and Nunoura 2016; Bonny et al. 2021; Geoghegan et al. 2021).

Members of the double-stranded RNA virus family *Picobirnaviridae* (order *Durnavirales*, class *Duplornaviricetes*, phylum *Pisuviricota*) have traditionally been considered opportunistic enteric pathogens of mammals and birds, typically detected in animal faecal samples (Malik et al. 2014), and were recently reported in invertebrate hosts (Delmas et al. 2019). In mammalian and avian species, *Picobirnavirus* infection has been associated with the disease presenting with diarrhoea or gastroenteritis (Ganesh, Masachessi, and Mladenova 2014; Malik et al. 2014). However, as picobirnaviruses have also been detected in the stool of healthy or asymptomatic animals (Ganesh, Masachessi, and Mladenova 2014), the presence of a *Picobirnavirus* in the animal gastrointestinal system is not necessarily associated with overt disease. The genomes of these picobirnaviruses are typically bi-segmented, with Segment 1 (2.4–2.7 kb in length) comprising three open reading frames (ORFs). ORF3 is known to code a capsid protein precursor, while recent evidence suggests that ORF1 and ORF2 may encode bacteriolytic proteins (Neri et al. 2022; Gan and Wang 2023). Segment 2 (1.7–1.9 kb) encodes the viral RNA-dependent RNA polymerase (RdRp) that is universal to RNA viruses and hence a powerful phylogenetic marker (Delmas et al. 2019). Picobirnaviruses with non-segmented genomes have also been reported, in which all ORFs are present in a single, monopartite genome (Giannitti et al. 2015; Shi et al. 2016).

The increasing accessibility of metatranscriptomic sequencing has resulted in the identification of huge numbers of *Picobirnavirus* sequences from both aquatic and terrestrial environmental samples such as wastewater, sewage, permafrost, farmland soils, and forest soils (Adriaenssens et al. 2018; Bell et al. 2020; Guajardo-Leiva et al. 2020; Chen et al. 2022; Neri et al. 2022). As metatranscriptomic sequencing necessarily produces a snapshot of all the RNA expressed in a sample, the host organisms for the majority of recently identified picobirnaviruses cannot be easily determined and characterisation of novel virus species largely relies on phylogenetic placement, often without the presence of complete virus genomes. While the number of *Picobirnavirus* and picobirna-like sequences available on the National Center for Biotechnology Information (NCBI)/GenBank is now in the thousands and comprises considerable genetic diversity, only a single genus and three species have been formally ratified in the most recent report of the International Committee on Taxonomy of Viruses (ICTV) (Delmas et al. 2019). The *Picobirnaviridae* are therefore in clear need of further taxonomic assessment and classification. An additional complicating factor is the large number of picobirnaviruses found within individual animal species, which has meant that multiple picobirnaviruses have been assigned the same name. For example, there are 12 viral RdRp sequences designated as ‘bovine picobirnavirus’, 13 as ‘dromedary picobirnavirus’, 16 as ‘human picobirnavirus’, and over 500 as ‘porcine picobirnavirus’ on NCBI/GenBank. Despite their identical names, these sequences are distinct and do not group together in phylogenetic trees (Delmas et al. 2019; Ghosh and Malik 2021).

The remarkably wide range of both animal and non-animal sources in which picobirnaviruses have been detected, combined with an ongoing inability to propagate these viruses in eukaryotic cell lines or detect them in solid tissue samples, has led to the proposal that the *Picobirnaviridae* are not exclusively animal-associated or that animals may not be the true hosts (Boros et al. 2018; Krishnamurthy and Wang 2018; Yinda et al. 2019; Kleymann et al. 2020; Ghosh and Malik 2021). The closest relative to the *Picobirnaviridae* is the plant-, fungi-, and protist-infecting family *Partitiviridae* (Vainio et al. 2018), members of which have also been detected in animal faeces (Chen et al. 2022) and invertebrate samples (Shi et al. 2016; Le Lay et al. 2020), suggesting that they infect components of animal diet rather than the animal themselves. Hence, it is reasonable to propose that the *Picobirnaviridae* are similarly associated with animal diet and/or commensal gut microflora. Aspects of *Picobirnavirus* genomes also support the hypothesis that these viruses do not infect animals (at least not exclusively) and may instead be associated with microbial organisms. In particular, the prokaryotic ribosomal binding site (RBS) motif, necessary for the initiation of translation in prokaryotes, is present in the genomes of other RNA bacteriophage such as in the *Cystoviridae* (Boros et al. 2018; Krishnamurthy and Wang 2018). A high prevalence of RBS motif sequences has been identified upstream of ORFs in both *Picobirnavirus* genome segments (Boros et al. 2018; Krishnamurthy and Wang 2018), suggesting that the picobirnaviruses, in fact, predominantly represent a family of bacteriophage. In addition, several *Picobirnavirus* species identified from mammals and invertebrates (Yinda et al. 2019; Kleymann et al. 2020) utilise the mitochondrial genetic code, a characteristic shared by members of the RNA virus family *Mitoviridae* (phylum *Lenarviricota*) that replicate in the fungal mitochondria (Cole et al. 2000; Hillman and Cai 2013).

Using a set of 2,286 publicly available *Picobirnaviridae* RdRp sequences, the majority of which were generated through metatranscriptomic sequencing, we aimed to provide a realistic classification of this important group of viruses, identifying distinct genera, and to reassess their host range. We based our analysis on the extent of congruence between virus and host phylogenetic trees, as well as on features of *Picobirnaviridae* genomes that may be indicative of bacterial hosts.

## Methods

### Data set collection and processing for phylogenetic analysis

For all phylogenies generated in this work, the number of sequences included in the data set, the outgroups used, and the lengths (trimmed and untrimmed) of each alignment generated are detailed in Table 1. The labels T1 through T21, where ‘T’ simply stands for ‘tree’, refer to an alignment or phylogeny generated on a particular combination of *Picobirnaviridae* and outgroup family sequences. The exception is T21, which is a host animal cladogram artificially generated in Newick format and used to assess host associations. The data sets assembled for each tree are detailed later.

**Table 1.** Details of sequence data sets, alignments, and rooting schemes used to generate phylogenetic trees.

		Alignment length (amino acid residues)			
Tree	Sequences analysed (total number)	Full length	Trimmed	Rooting scheme	Figure(s)
T1	*Picobirnaviridae* (2,286)	13,540	704	Midpoint	Fig. 1A
T2	*Picobirnaviridae* (2,286), *Amalgaviridae* (14)	13,430	712	*Amalgaviridae*	Fig. 1B
T3	*Picobirnaviridae* (2,286), *Curvulaviridae* (8)	13,744	701	*Curvulaviridae*	Fig. 1C
T4	*Picobirnaviridae* (2,286), *Fusariviridae* (32)	13,738	701	*Fusariviridae*	Fig. 1D
T5	*Picobirnaviridae* (2,286), *Partitiviridae* (2,536)	18,017	757	*Partitiviridae*	Fig. 1E
T6	*Picobirnaviridae* (2,286), *Partitiviridae* (2,536), *Amalgaviridae* (14)	17,740	710	*Amalgaviridae*	Fig. 1F
T7	*Picobirnaviridae* (2,286), *Partitiviridae* (2,536), *Curvulaviridae* (8)	17,742	710	*Curvulaviridae*	Fig. 1G
T8	*Picobirnaviridae* (2,286), *Partitiviridae* (2,536), *Fusariviridae* (32)	17,231	706	*Fusariviridae*	Fig. 1H
T9	*Picobirnaviridae* (2,286), *Partitiviridae* (40)	13,906	695	*Partitiviridae*	Fig. 1I, Fig. 4B, D, Supplementary Fig. 9, 11
T10	*Picobirnaviridae* (2,286), *Partitiviridae* (394)	14,063	703	*Partitiviridae*	Fig. 1J
T11	*Durnavirales* excluding family *Hypoviridae* (4,717)	14,971	808	Unrooted	Fig. 2
T12	‘Alphapicobirnavirus’ (421)	3,639	728	Midpoint	Fig. 3A, Supplementary Fig. 1
T13	‘Betapicobirnavirus’ (802)	2,844	711	Midpoint	Fig. 3B, Supplementary Fig. 2
T14	‘Gammapicobirnairus’ (418)	3,856	733	Midpoint	Fig. 3E, Supplementary Fig. 5
T15	‘Deltapicobirnavirus’ (311)	5,759	749	Midpoint	Fig. 3F, Supplementary Fig. 6
T16	‘Epsilonpicobirnavirus’ (36)	853	691	Midpoint	Fig. 3C, Supplementary Fig. 3
T17	‘Zetapicobirnavirus’ (67)	824	700	Midpoint	Fig. 3D, Supplementary Fig. 4
T18	‘Etapicobirnavirus’ (70)	881	705	Midpoint	Fig. 3G, Supplementary Fig. 7
T19	Animal-associated *Picobirnaviridae* (1,269), *Partitiviridae* (40)	5,734	745	*Partitiviridae*	Fig. 4A, C, Supplementary Figs. 8, 10
T20	Animal-associated *Picobirnaviridae* (1,269)	5,591	727	Midpoint	Input for Fig. 5 (not shown)
T21	NA—artificially generated host cladogram	NA	NA	NA	Input for Fig. 5, Supplementary Fig. 12

As a base *Picornaviridae* data set, curated in June 2022, we collated all non-redundant *Picobirnavirus* RdRp sequences available on NCBI/GenBank by searching the protein database for ‘picobirna’. Only sequences with associated hosts or sampling environment metadata were included. *Picobirnavirus* and picobirna-like sequences described by Neri et al. (2022) were also added to this data set. The majority (89 per cent) of sequences collected were faecal or environmentally sourced picobirnaviruses described in Chen et al. (2022) and Neri et al. (2022). A total of 3,644 sequences clustered at 90–99 per cent amino acid identity using the Cluster Database at High Identity with Tolerance program (Fu et al. 2012). One representative sequence from each cluster was retained. The total number of *Picobirnavirus* sequences collated in this manner for further analysis was 2,286.

RdRp sequences from families related to the *Picobirnaviridae* within the order *Durnavirales* were included in additional alignments as outgroups to provide directionality to the resulting phylogenies: the *Amalgaviridae* (14 sequences; T2 and T6 in Table 1), *Curvulaviridae* (8 sequences; T3 and T7), *Fusariviridae* (32 sequences; T4 and T8), and the *Partitiviridae* (40, 394, or 2,536 sequences; T9, T10, and T5–8, respectively). A phylogeny of the order *Durnavirales* was estimated on 4,717 sequences from the families *Amalgaviridae*, *Curvulaviridae*, *Fusariviridae*, *Picobirnaviridae*, and *Partitiviridae* (T11). Phylogenetic trees were also estimated based on sequence alignments of each of the proposed *Picobirnaviridae* genera (T12–18). Finally, sequence alignments were constructed on a subset of 1,269 *Picobirnavirus* sequences detected in animal (typically faecal) samples, with (T19) and without (T20) an outgroup of 40 partitivirus sequences.

### Sequence alignment and phylogenetic analysis

All sequences were aligned using MAFFT (v7.487) (Katoh and Standley 2013) and trimmed using trimAL (v1.4.1) (Capella-Gutierrez, Silla-Martinez, and Gabaldon 2009) to retain the most conserved amino acid positions. Trimmed alignments were manually inspected in Geneious Prime 2022.2.1 to identify and remove any ambiguously aligned regions, resulting in trimmed alignments ranging between 691 and 757 positions in length used to generate phylogenetic trees.

The best-fit amino acid substitution model for all data sets was found to be the Le–Gascuel (LG) model, established using ModelFinder within IQ-TREE (v1.6.2). Due to the consistently very high number of amino acid changes per position, a gamma distribution of among-site rate variation was not used to estimate the full *Durnavirales* phylogeny (T11 in Table 1). Maximum likelihood phylogenetic trees were then estimated on each data set using IQ-TREE (v1.6.2) (Nguyen et al. 2015), with node support evaluated using the SH-like approximate likelihood ratio test (SH-aLRT), with 1,000 replicates. Trees were visualised in FigTree (v1.4.4) (Rambaut 2018), as well as in R (v4.1.0) using the packages ‘ape’ (v5.5) (Paradis, Schliep, and Schwartz 2019) and ggtree (v3.0.2) (Yu et al. 2017).

### Classification of the Picobirnaviridae

Ten phylogenies (T1–T10; see Table 1) were used for determining potential genera within the *Picobirnaviridae*. Four large and three small defined clusters were identified in the midpoint-rooted tree (T1 in Table 1), defined by relatively long branch lengths to their respective common ancestor node and ≥80 per cent SH-aLRT support. Sequences within each cluster were annotated as ‘clade 1’ through ‘clade 7’. These clusters or ‘draft genera’ were then annotated in all other trees. Any sequences not clustering within their assigned ‘draft genus’ with ≥80 per cent SH-aLRT node support in any of the ten trees were excluded. The remaining sequences that consistently grouped within the same clade in every tree were considered the ‘core’ sequences of each proposed genus. Sequence alignments were constructed on each set of core sequences, and subsequent genus-level phylogenetic trees were estimated as described earlier.

### Analysis of phylogenetic incongruence

Prior to analysis, *Picobirnaviridae* sequences were grouped by assigned host (animals) or sampling source (environments and microbial communities). Hosts and sampling sources were categorised in two ways (Table 2). The first focused on specific animal hosts, with non-animal sources simply reflected in a joint ‘microbial/environmental’ category, while mammals were further categorised into lower taxonomic levels. The second grouped animal hosts more broadly as ‘mammalian’, ‘avian’, ‘fish’, ‘reptile’, and ‘invertebrate’, with non-animal sources similarly expanded into ‘aquatic’, ‘terrestrial’, and ‘engineered’ environments and microbial communities. Aquatic sources included bodies of water and sediments, while terrestrial environments included various soils and plant matter (Chen et al. 2022; Neri et al. 2022). Engineered sources included wastewater, food fermentation processes, and laboratory cultures (Neri et al. 2022).

**Table 2.** Categories of assumed host animal or environmental/microbial source of the *Picobirnaviridae* sequences analysed in this study.

Animal-host focused list	Environmental/microbial source-focused list
Primate	Mammalian
Bovidae	
Cervidae	
Equidae	
Suidae (porcine)	
Dromedary	
Feline	
Canine	
Tasmanian devil	
Rodent	
Lagomorph	
Bat	
Avian	Avian
Reptile	Reptile
Fish	Fish
Arthropod	Invertebrate
Other invertebrate	
Microbial/environmental	Microbial—aquatic
	Microbial—engineered
	Microbial—terrestrial
	Environmental—aquatic
	Environmental—engineered
	Environmental—terrestrial

A cladogram of relevant ‘host’ animals (as listed in Table 2) was constructed based on the evolutionary relationships demonstrated in the current literature on vertebrate and invertebrate evolution (Lindblad-Toh et al. 2005; Fong et al. 2012; Song et al. 2012; Shen et al. 2014; Prum et al. 2015; Zhou, Wang, and Ma 2017; Zurano et al. 2019). The eMPRess program (v1.2.1) (Santichaivekin et al. 2021) was used to reconcile topologies of the animal-associated *Picobirnavirus* (T20 in Table 1) and host animal (T21 in Table 1) phylogenies to determine the event likelihoods of co-divergence, cross-species transmission, duplication, and extinction. Event costs were defined as 0 for co-divergence and 1 for cross-species transmission, duplication, and extinction. The R package ‘NELSI’ (v0.21) (Ho, Duchêne, and Duchêne 2015) was used to calculate the normalised PH85 (nPH85) distance for the *Picobirnaviridae*. The nPH85 distance is based on Penny and Hendy distance metric and describes the topological distance between the virus and host phylogenies (Geoghegan, Duchêne, and Holmes 2017). An nPH85 distance of 0 indicates complete co-divergence between virus and host, whereas an nPH85 of 1 indicates complete cross-species transmission.

### Presence of bacterial RBSs

A sequence alignment was generated using MAFFT (v7.487) comprising all available non-redundant and annotated nucleotide sequences of Segment 1 of the *Picobirnavirus* genome that contained at least 24 nucleotides of a 5ʹ untranslated region (UTR), totalling 352 sequences. Sequences were aligned to assist in locating the start codons of ORFs, upstream of which the RBS motifs are potentially located. Similarly, an alignment was constructed comprising all available Segment 2 *Picobirnaviridae* sequences using the same criteria, for a total of 984 sequences. The twenty-four nucleotides upstream of the start codon of each annotated ORF were annotated and extracted using Geneious Prime 2022.2.1. Occurrences of the ‘AGGAGG’ hexamer, ‘AGGAG’ or ‘GGAGG’ 5-mers, or ‘AGGA’, ‘GGAG’ or ‘GAGG’ 4-mers within each extracted 24-nucleotide region were identified and annotated using Geneious Prime 2022.2.1, as these likely represented a bacterial RBS motif and hence are evidence of a bacterial host. The frequency of RBS occurrence for each segment was calculated as a proportion of the total number of annotated ORFs in the set of sequences for that segment. To compare the frequencies of RBS presence between the *Picobirnaviridae* and established RNA bacteriophage families, this process was repeated on 1,040 *Leviviricetes* genomes and 17, 11, and 38 *Cystoviridae* S, M, and L segments, respectively.

## Results

### Classification of the Picobirnaviridae

**Figure 1.**
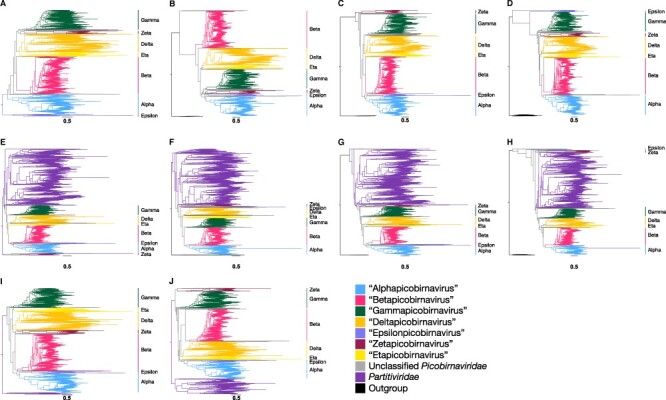
Maximum likelihood phylogenies of the family *Picobirnaviridae* estimated on the RdRp and using different families of the order *Durnavirales* as outgroups. Row one contains the *Picobirnaviridae*: (A) midpoint-rooted or utilising the (B) *Amalgaviridae*, (C) *Curvulaviridae*, or (D) *Fusariviridae* as outgroups. Row two contains the multi-family *Partitivirdae*–*Picobirnaviridae* clade: (E) midpoint-rooted or utilising the (F) *Amalgaviridae*, (G) *Curvulaviridae*, or (H) *Fusariviridae* as outgroups. Row three contains the *Picobirnaviridae* with a subset of (I) 40 or (J) 394 *Partitiviridae* sequences as outgroups. In all trees, the proposed *Picobirnaviridae* genera—‘Alpha-’, ‘Beta-’, ‘Gamma-’, ‘Delta-’, ‘Epsilon-’, ‘Zeta-’, and ‘Etapicobirnavirus’*—*are labelled. Horizontal branches are drawn to scale, and the scale bars below each tree represent 0.5 amino acid substitutions per site.

We observed seven major well-supported clusters of RdRp sequences within the *Picobirnaviridae* that were consistently present across ten phylogenies inferred using different outgroups and rooting methods (Fig. 1, Table 1). Each of the seven clades were defined by relatively long branches to their respective common ancestral node with >80 per cent SH-aLRT node support in each of the ten phylogenies (Fig. 1). We propose that these groups are candidates for novel genera with the *Picobirnaviridae*, tentatively named: ‘Alphapicobirnavirus’ (421 sequences, containing *Orthopicobirnavirus equi*), ‘Betapicobirnavirus’ (802 sequences, containing *Orthopicobirnavirus hominis*), ‘Gammapicobirnavirus’ (418 sequences, containing *Orthopicobirnavirus beihaiense*), ‘Deltapicobirnavirus’ (311 sequences), ‘Epsilonpicobirnavirus’ (36 sequences), ‘Zetapicobirnavirus’ (67 sequences), and ‘Etapicobirnavirus’ (70 sequences), in line with the nomenclature for other multi-genus families within the *Durnavirales*. While these are ‘proposed’ or ‘candidate genera’ only and will not be italicised following ICTV convention, for simplicity they will be hereafter referred to directly as ‘genera’. Sequences consistently clustering within one of these clades in each of the ten trees were considered the ‘core’ members comprising each genus. Those sequences whose phylogenetic position was inconsistent remained as ‘unclassified’ *Picobirnaviridae*. Using this approach, we were able to assign 2,125 non-redundant picobirnaviruses to a proposed genus (Supplementary Table 1). Genus-level phylogenies of each proposed genus are shown in Supplementary Figures 1–7.

The only currently accepted genus in the *Picobirnaviridae*—*Orthopicobirnavirus—*contains three species: *Orthopicobirnavirus hominis*, *Orthopicobirnavirus equi*, and *Orthopicobirnavirus beihaiense*, isolated from human (Wakuda, Pongsuwanna, and Taniguchi 2005), horse (Giannitti et al. 2015), and peanut worms (Shi et al. 2016), respectively. Each of these species appear in a different proposed genus described here (Supplementary Figures 1–3), limiting our ability to clearly determine which most closely resembles the genus *Orthopicobirnavirus*.

There were no common topologies for the proposed *Picobirnaviridae* genera across all ten phylogenetic trees. As a consequence, exact evolutionary relationships among genera cannot be safely determined. However, the genera ‘Deltapicobirnavirus’ and ‘Etapicobirnavirus’ formed sister clades in seven of the ten phylogenies (Fig. 1A–D, F, H, J). In the remaining three trees (Fig. 1E, G, I), one genus fell directly basal to the other, but which genus was basal to the other was inconsistent among trees. In six phylogenies, ‘Alphapicobirnavirus’ and ‘Epsilonpicobirnavirus’ formed sister clades (Fig. 1B, C, E, G, I, J), suggesting that these genera are more closely related to each other. In eight trees, the two largest genera, ‘Alphapicobirnavirus’ and ‘Betapicobirnavirus’, either grouped together (Fig. 1D–I) or one fell directly basal to the other (Fig. 1A, C). Furthermore, in four of the trees where ‘Alphapicobirnavirus’ and ‘Betapicobirnavirus’ genera clustered together, ‘Epsilonpicobirnavirus’ was also part of the group as a sister clade to ‘Alphapicobirnavirus’ (Fig. 1C, E, G, I). Based on these topological patterns, we further suggest that subfamilies may also eventually be defined, likely one comprising ‘Alpha-’, ‘Beta-’, and ‘Epsilonpicobirnavirus’, with another comprising ‘Delta-’ and ‘Etapicobirnavirus’. However, more sequences are required to achieve a more consistent topology before such subfamilies can be confidently defined.

**Figure 2.**
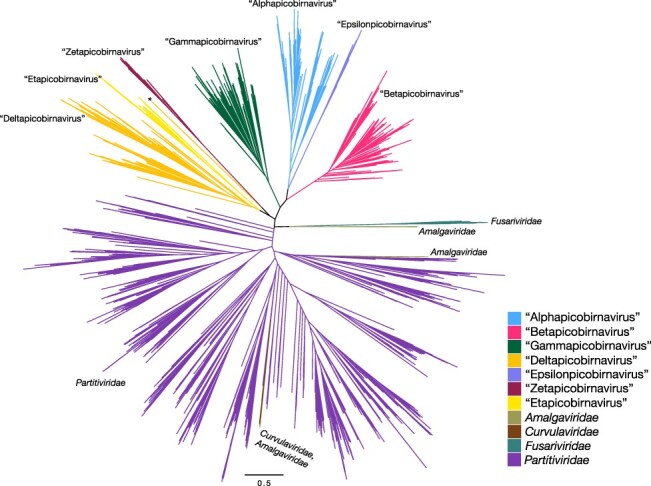
Unrooted maximum likelihood tree of 4,717 *Durnavirales* RdRp sequences, with branches labelled and coloured by family (*Amalgaviridae, Curvulaviridae, Fusariviridae*, and *Partitiviridae*) or proposed genus (*Picobirnaviridae*), namely ‘Alpha-’, ‘Beta-’, ‘Gamma-’, ‘Delta-’, ‘Epsilon-’, ‘Zeta-’, and ‘Etapicobirnavirus’. The star represents a clade of seven divergent deltaviruses that clustered outside of the proposed genus. Branch lengths indicate the number of amino acid substitutions per site, as represented by the scale bar.

We next sought to give these proposed genera evolutionary context by placing them in a tree with multiple related families of RNA viruses. An unrooted phylogeny was estimated on 4,717 RdRp sequences from the order *Durnavirales*, including the *Picobirnaviridae, Partitiviridae, Amalgaviridae, Curvulaviridae*, and *Fusariviridae* (Fig. 2). Sequences from the family *Hypoviridae* were too divergent to reliably align and were therefore excluded. Importantly, the *Picobirnaviridae* formed a monophyletic group that was phylogenetically distinct from the other families in the order. Certain trends in tree topology frequently observed in Fig. 1 were repeated here, such as ‘Deltapicobirnavirus’ and ‘Etapicobirnavirus’ forming sister clades, as well as ‘Alphapicobirnavirus’ and ‘Epsilonpicobirnavirus’ forming sister clades that clustered with ‘Betapicobirnavirus’ (Fig. 2). The proposed genera also remained mostly intact despite the high level of diversity in the alignment. The only exception was a group of seven divergent deltapicobirnaviruses that fell as a small sister clade to the zetapicobirnaviruses (Fig. 2, denoted by a star).

### Distribution of apparent hosts or environmental source of picobirnaviruses in each proposed genus

**Figure 3.**
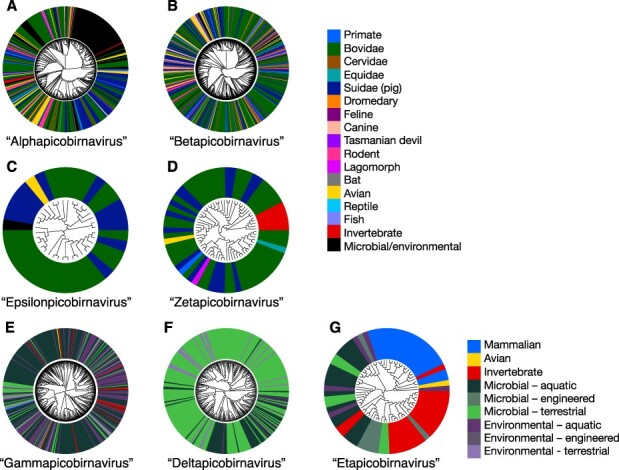
Maximum likelihood phylogenetic trees of the RdRp of proposed *Picorbirnaviridae* genera coloured by apparent host or sampling environment. All phylogenies are midpoint-rooted. Tip labels are coloured to represent assigned host/sampling environment. ‘Alphapicobirnavirus’ (A), ‘Betapicobirnavirus’ (B), ‘Epsilonpicobirnavirus’ (C), and ‘Zetapicobirnavirus’ (D) are coloured by a specific assigned animal host. ‘Gammapicobirnavirus’ (E), ‘Deltapicobirnavirus’ (F), and ‘Etapicobirnavirus’ (G) categorise animal hosts more broadly as ‘mammalian’, ‘avian’, and ‘invertebrate’, and non-animal-associated sequences are grouped into ‘environmental’ or ‘microbial’ samples, both further classed into ‘terrestrial’, ‘aquatic’, and ‘engineered’ sampling sources. Branch lengths indicate the number of amino acid substitutions per site. Scale bars for branch lengths are shown in rectangular versions of trees (Supplementary Figures 1–7).

The genus ‘Alphapicobirnavirus’ (Fig. 3A, Supplementary Figure 1) predominantly comprised mammalian and avian picobirnaviruses, as well as those sampled from some invertebrate species. In the most basal clade of this genus, the majority of sequences were identified in microbial communities, although a small number of mammalian, avian, invertebrate, and environmentally sourced picobirnaviruses also fell in this group. The genus ‘Betapicobirnavirus’ (Fig. 3B, Supplementary Figure 2) was similarly dominated by picobirnaviruses from mammalian and avian sources. This genus also contained the divergent species identified in fish (AVM87403 Beihai goldsaddle goldfish *Picobirnavirus*) and lizards (AVM87436 Guangdong Chinese water skink *Picobirnavirus* and UCS96434 *Picobirnaviridae* sp.). There were fewer microbial/environmental-sourced viruses in the betapicobirnaviruses and none formed large, monophyletic clusters as seen in the basal ‘Alphapicobirnavirus’ group.

In contrast to the predominantly animal-associated genera described earlier, the ‘Gammapicobirnavirus’ (Fig. 3E, Supplementary Figure 3) and ‘Deltapicobirnavirus’ (Fig. 3F, Supplementary Figure 4) genera were almost entirely composed of sequences detected in microbial communities and environmental samples. Some invertebrate-associated picobirnaviruses (namely, from Cnidara, Porifera, crustaceans, and molluscs) clustered within the ‘Gammapicobirnavirus’ (Supplementary Figure 3), as well as two picobirnaviruses from vertebrate faecal samples (USE08169 *Picobirnavirus* sp. from pig faeces and QUS52969 mute swan faeces associated *Picobirnavirus* 3). However, most viruses from this genus were sampled in environmental or microbial samples, particularly aquatic sources.

Viruses in the genus ‘Deltapicobirnavirus’ were predominantly sourced from terrestrial microbe communities and environments (Fig. 3F, Supplementary Figure 4). The most recently diverged clade had a more even distribution of viruses sampled from aquatic and terrestrial environments and microbes (Supplementary Figure 4). Again, two picobirnaviruses sourced from animals were present in this genus—ND_241614 and ND_192065—from cattle and sheep, respectively. However, these sequences were mined from animal rumen microbiome sequence data (Neri et al. 2022), thereby making ‘Deltapicobirnavirus’ the only genus containing entirely microbe-associated or environmentally sourced viruses.

The remaining genera were considerably smaller. All but one of the thirty-six viruses falling into the genus ‘Epsilonpicobirnavirus’ were sourced from vertebrate faeces, the majority of which were mammalian (cattle and porcine) (Fig. 3C, Supplementary Figure 5). The genus ‘Zetapicobirnavirus’ also featured a similar range of sampling sources, with the majority being cattle and porcine samples, along with one avian- and one primate-sourced *Picobirnavirus*. This phylogeny included a notable divergent clade of five arthropod-associated zetapicobirnaviruses (Fig. 3D, Supplementary Figure 6). Finally, despite only comprising seventy sequences, the genus ‘Etapicobirnavirus’ had a broader ‘host’ range across the two sister clades observed within this group. One predominantly comprised vertebrate etapicobirnaviruses, as well as one sequence identified in a termite that is likely associated with its bacterial symbionts (Fig. 3G, Supplementary Figure 7). The other clade included another 15 etapicobirnaviruses identified in termite samples, with the basal lineages largely viruses sourced from microbial and aquatic environmental samples (Supplementary Figure 7).

According to published information, picobirnaviruses utilising an alternative genetic code appeared in the ‘Alphapicobirnavirus’ (one sequence), ‘Betapicobirnavirus’ (six sequences), ‘Gammapicobirnavirus’ (one sequence), and ‘Zetapicobirnavirus’ (six sequences) proposed genera. Of the six betapicobirnaviruses, one utilised the flatworm mitochondrial code (translation table 14), while the remainder used the ciliate code (translation table 6), although none of these sequences clustered together (Supplementary Figure 2). This is in contrast to the zetapicobirnaviruses, where all viruses utilising alternative genetic codes fell in the basal lineages (Supplementary Figure 6). One used the ciliate genetic code, while the other five were all arthropod-associated picobirnaviruses using the invertebrate mitochondrial code (translation table 5) and formed a monophyletic group. The single sequences within ‘Alpha-’ and ‘Gammapicobirnavirus’ that utilised an alternative genetic code shared only 48 per cent and 40 per cent amino acid identity with their nearest relatives, respectively. This suggests a large amount of missing diversity within these clades and that many more picobirnaviruses utilising alternative genetic codes may be identified in future studies.

### Accuracy of host assignments based on phylogenetic position

**Figure 4.**
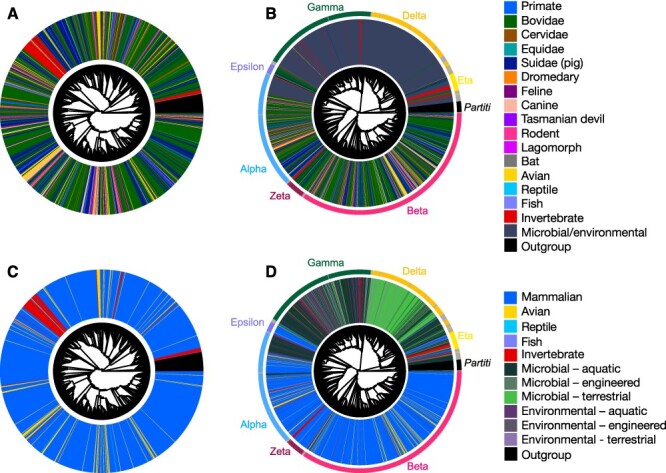
Maximum likelihood trees of *Picobirnaviridae* RdRp with tips coloured to represent assigned host/sampling environment. *Partitiviridae* RdRp sequences (*n* = 40) were used to root all trees and are shown in black. The top two trees are coloured by animal host groups, including lower-level Mammalia groupings, and are estimated on (A) *Picobirnaviridae* sequences with assumed animal associations and (B) all *Picobirnaviridae* sequences. The bottom two trees categorise animal hosts more broadly as ‘mammalian’, ‘avian’, ‘reptile’, ‘fish’, and ‘invertebrate’ and are estimated using (C) *Picobirnaviridae* sequences with assumed animal associations and (D) all *Picobirnaviridae* sequences with non-animal-associated sequences grouped into ‘environmental’ or ‘microbial’ samples, both further classed into ‘terrestrial’, ‘aquatic’, and ‘engineered’ sampling sources. Scale bars for branch lengths are shown in rectangular versions of trees (Supplementary Figures 8–11).

To visualise the extent of apparent cross-species transmission within the *Picobirnaviridae* based on assigned hosts/sample source, we estimated a phylogeny of only the animal-sourced picobirnaviruses (Fig. 4A, T19 in Table 1) and a complete *Picobirnavirus* phylogeny (Fig. 4B, T9 in Table 1). Both trees were rooted by a set of forty partitiviruses as they represent the most closely related family to the *Picobirnaviridae*. The diverse distribution of assigned host species across the phylogeny, as demonstrated in Fig. 4A and B where tips are coloured by host, revealed a lack of consistent clustering by assigned host. Even at the order level within the *Mammalia*, closely related virus sequences had assigned hosts spanning diverse groups of mammals. Picobirnaviruses from birds did not form any large monophyletic groups, and instead clustered with species from cattle, pigs, felines, canines, primates, and marsupials (Fig. 4A, C). This was also clearly observable within the predominantly animal-associated genera ‘Alphapicobirnavirus’, ‘Betapicobirnavirus’, ‘Epsilonpicobirnavirus’, and ‘Zetapicobirnavirus’ (Fig. 3A–D, Supplementary Figures 1–4). Although microbial/environmentally sourced viruses tended to group together in clades distinct from the animal-sourced picobirnaviruses, there were several invertebrate-, primate-, cattle-, pig-, and avian-associated species present in the environmental/microbial clades (Fig. 4B, D). Notably, representatives from all subcategories of microbial and environmental picobirnaviruses were present in animal-associated clades with the exception of those sourced from terrestrial microbes (Fig. 4D). Rectangular, midpoint-rooted versions of the trees shown in Fig. 4 with tip labels including accession numbers and virus names are shown in Supplementary Figures 8–11. The distribution of assigned hosts and sampling sources across each genus is shown in Supplementary Figure 12.

**Figure 5.**
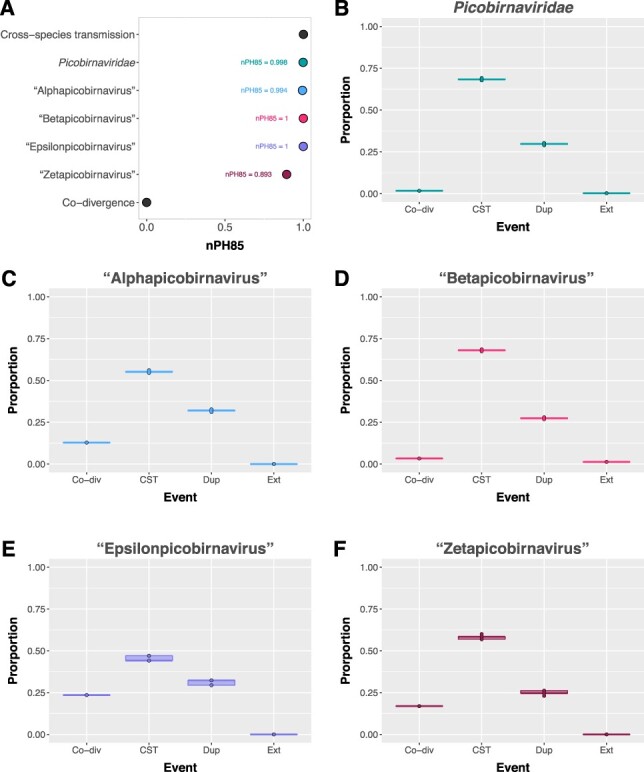
Levels of co-divergence of the *Picobirnaviridae* with associated animal hosts. (A) nPH85 metric calculated for the *Picobirnaviridae*, ‘Alphapicobirnavirus’, ‘Betapicobirnavirus’, ‘Epsilonpicobirnavirus’, and ‘Zetapicobirnavirus’ using NELSI, representing the topological distance between unrooted virus and host trees. nPH85 = 0 indicates complete co-divergence, and nPH85 = 1 indicates complete cross-species transmission. Event likelihoods of co-divergence (Co-div), cross-species transmission (CST), duplication (Dup), and extinction (Ext) as a proportion of all possible events in the reconciled co-phylogenies of animal-associated *Picobirnavirus* sequences and their assigned hosts for (B) the family *Picobirnaviridae*, and the genera (C) ‘Alphapicobirnavirus’, (D) ‘Betapicobirnavirus’, (E) ‘Epsilonpicobirnavirus’, and (F) ‘Zetapicobirnavirus’. Event likelihoods were calculated in eMPRess with 100 replicates.

A phylogeny of animal-associated picobirnaviruses (T20 in Table 1) was compared to a host phylogeny (Supplementary Figure 13, T21 in Table 1) using NELSI (Ho, Duchêne, and Duchêne 2015) to estimate the frequency of cross-species transmission (assuming assigned hosts are the true hosts) by calculating the nPH85 metric that describes the topological distance between the two phylogenies. The nPH85 distance between the unrooted animal-associated *Picobirnaviridae* tree and host taxa tree was 0.998 (Fig. 5A), suggesting almost complete cross-species transmission and very little occurrence of co-divergence among the animal-associated members of this family. Similarly, the nPH85 distance for the genus ‘Alphapicobirnavirus’ was 0.994 and 1 for both the ‘Betapicobirnavirus’ and ‘Epsilonpicobirnavirus’. The genus ‘Zetapicobirnavirus’ had a comparatively higher, yet still low, level of co-divergence with an nPH85 distance of 0.921 (Fig. 5A). eMPRess (Santichaivekin et al. 2021) was utilised to determine the respective likelihoods of co-divergence, cross-species transmission, duplication, or extinction in the reconciled virus–host co-phylogeny. The event likelihoods are shown in Fig. 5B–F and reveal that cross-species transmission was far more frequent than any other event (68 per cent of events, Fig. 5B) for the entire family as well as for each individual genus (45–68 per cent of events, Fig. 5C–F). In contrast, only low levels of apparent virus–host co-divergence were observed, especially at the level of the entire *Picobirnaviriade* (1–23 per cent of events, Fig. 5B–F).

### Genetic evidence of *Picobirnaviridae* in non-animal hosts

We next searched for bacterial RBS motifs in a data set of 1,336 *Picobirnavirus*, 1,040 *Levivirus*, and 64 *Cystovirus* genome segments. The detection of an ‘AGGAGG’ hexamer up to twenty-four nucleotides upstream of the start codon of an annotated ORF was considered to indicate the presence of a full RBS motif. Partial RBS motifs included the 4-mers AGGA, GGAG, and GAGG, and the 5-mers AGGAG and GGAGG. There were 687 annotated ORFs across the 352 *Picobirnavirus* Segment 1 nucleotide sequences and 782 annotated ORFs across the 984 *Picobirnavirus* Segment 2 sequences. The number of Segment 1 and Segment 2 ORFs preceded by a full or partial RBS motif was 571 (83.1 per cent) and 667 (85.3 per cent), respectively (Table 3). Strikingly, this was a higher proportion of RBS motif enrichment than observed in the confirmed RNA bacteriophage groups of *Leviviricetes* (1901 of 3251 ORFs; 58.5 per cent) and *Cystoviridae* (169 of 294 ORFs; 57.1 per cent) (Table 3). This difference was most prominent with respect to the frequency of a complete RBS motif: 52.8 per cent and 69.3 per cent of ORFs in *Picobirnaviridae* genome Segments 1 and 2, respectively, were preceded by the ‘AGGAGG’ motif, compared to 0–6.4 per cent of ORFs in *Cystoviridae* or *Leviviricetes* sequences. In contrast, *Picobirnaviridae* genomes segments had much lower frequencies of 4-mer partial RBS motifs (5.4–5.8 per cent) compared to 13.7–33.5 per cent of *Cystoviridae* or *Leviviricetes* ORFs (Table 3).

**Table 3.** Genome segments and calculated proportions of annotated ORFs with the full ‘AGGAGG’ RBS motif or a 4-mer or 5-mer partial RBS motif up to twenty-four nucleotides upstream of the start codon.

Genome segment	Number of nucleotide sequences	Number of annotated ORFs	Total number of RBS motifs^a^ (full or partial)	4-mer^a^ (AGGA| GGAG| GAGG)	5-mer^a^ (AGGAG| GGAGG)	6-mer^a^ (AGGAGG)
*Picobirnavirus* Segment 1	352	687	571 (83.1)	37 (5.4)	171 (24.9)	363 (52.8)
*Picobirnavirus* Segment 2	984	782	667 (85.3)	45 (5.8)	80 (10.2)	542 (69.2)
*Leviviricetes* genome	1040	3219	1901 (58.5)	938 (28.9)	758 (23.3)	205 (6.3)
*Cystovirus* S segment	15	76	30 (39.5)	25 (32.9)	5 (6.6)	0 (0)
*Cystovirus* M segment	11	51	21 (41.2)	7 (13.7)	12 (23.5)	2 (3.9)
*Cystovirus* L segment	38	167	117 (70.1)	56 (33.5)	54 (32.3)	7 (4.2)

a Values in parentheses indicate the percentage of annotated ORFs preceded by an RBS motif.

## Discussion

The *Picobirnaviridae* are a family of RNA viruses traditionally associated with opportunistic gastroenteritis in humans and other animals that have dramatically increased in size and diversity in recent years due to metatranscriptomic sequencing. Despite this marked increase in diversity, picobirnaviruses remain largely unclassified beyond the family level, with only three divergent species comprising a single genus having been formally accepted by the ICTV (Delmas et al. 2019). The central aim of this study was to provide a taxonomic structure for this family by identifying distinct clades within the *Picobirnaviridae* that may represent genera (while noting that all such definitions are arbitrary). To this end, multiple outgroups and rooting schemes were utilised in an expansive phylogenetic analysis. From this, we identified seven clusters of sequences that consistently appeared across ten different phylogenies, which we propose be considered as distinct genera: ‘Alphapicobirnavirus’, ‘Betapicobirnavirus’, ‘Gammapicobirnavirus’, ‘Deltapicobirnavirus’, ‘Epsilonpicobirnavirus’, ‘Zetapicobirnavirus’, and ‘Etapicobirnavirus’. In addition, these seven proposed genera remained mostly intact and together formed a monophyletic, phylogenetically distinct group within the order *Durnavirales*. No genera shared greater than an average of 19 per cent amino acid identity in the RdRp with any other genera, which is in line with the <24 per cent amino acid identity shared between genera of the related *Partitiviridae* (Vainio et al. 2018).

Correctly assigning host organisms to novel virus species is a major limitation of the meta-transcriptomic approach to virus discovery. While the *Picobirnaviridae* have historically been associated with humans and other animals, there is mounting evidence that animals may not be the true hosts (Ghosh and Malik 2021). As a consequence, we asked whether the *Picobirnaviridae* were a true animal-infecting family of RNA viruses or one that infect microbiota themselves associated with animal hosts, i.e. that they are bacteriophage or other microbe-infecting viruses. The marked lack of topological congruence between phylogenies of the animal-associated picobirnaviruses and their apparent animal hosts indicates that the evolutionary history of the *Picobirnaviridae* has been characterised by extensive host-jumping, with statistical tests revealing near complete cross-species transmission. Notably, the nPH85 distance for animal-associated picobirnaviruses (0.998) is higher than the average for RNA virus families (0.95) and indeed higher than *Rhabdoviridae* (0.989), the family with the highest nPH85 distance of those analysed in Geoghegan, Duchêne, and Holmes (2017). This supports previous suggestions that animal-associated picobirnaviruses do not cluster according to their ‘host’ of sampling (Duraisamy et al. 2018; Woo et al. 2019; Mahar et al. 2020), although the current study uses a much larger data set. The complete lack of apparent host and viral phylogenetic congruence points towards an error in host assignment rather than the less biologically likely alternative of near complete cross-species transmission at a rate not seen in any other RNA virus family.

Picobirnaviruses have been detected in a large range of animal species, including those from terrestrial and aquatic habitats, invertebrates and vertebrates and those with herbivorous, carnivorous and omnivorous diets. This raises the question of how picobirnaviruses are able to overcome the geographical and biological barriers between animals with remarkably different habitats, lifestyles, and gastrointestinal systems, if they truly infect the hosts being sampled. Broad host ranges spanning vertebrates and invertebrates have been observed in several RNA virus orders, such as the *Articulavirales, Nidovirales, Reovirales*, and *Mononegevirales*, with the latter two also carrying fungi-infecting species. At a family level, the *Orthomyxoviridae, Rhabdoviridae*, and *Spinareoviridae* also infect a wide variety of hosts, including mammals, birds, insects, and other arthropods, along with fish in the case of the *Orthomyxoviridae*, plants in the *Rhabdoviridae*, and fungi in the *Spinareoviridae*. There are, however, clear phylogenetic distinctions between genera infecting vastly different hosts, and host ranges are generally limited at a genus level. Genera with cross-phylum or cross-kingdom host ranges are typically characterised by well-established transmission routes between arthropod vectors and animal or plant hosts. For example, arboviruses in the genera *Orthoflavivirus, Alphavirus, Coltivirus*, and *Orbivirus* replicate in their vertebrate hosts as well as the arthropod vectors that transmit them (Huang et al. 2023). Similarly, plant-infecting genera in the families *Tospoviridae, Tymoviridae* (*Marafivirus*), *Rhabdoviridae*, and order *Reovirales* also replicate in their arthropod vectors (Gray and Banerjee 1999). While it is plausible that members of the *Picobirnaviridae* are capable of extensive host-jumping, their remarkably wide host range raises the possibility that the picobirnaviruses are in fact associated with gut microflora or dietary components present in and excreted by these animals (Krishnamurthy and Wang 2018). This would also explain why non-environmental picobirnaviruses are detected almost exclusively in animal faeces and are still unable to be cultured in any eukaryotic cell lines (Ghosh and Malik 2021).

The presence of a bacterial RBS motif is well established in bacteriophage genomes. Krishnamurthy and Wang (2018) noted that the ‘AGGAGG’ RBS hexamer is enriched in the genomes of both RNA and DNA bacteriophage and appears at much lower frequency in eukaryote-infecting virus genomes. In our expansive data set, we detected an RBS motif in 83–85 per cent of ORFs in *Picobirnavirus* genomes, such that this marker is substantially more enriched than in confirmed RNA bacteriophages *Leviviricetes* and *Cystoviridae*. This constitutes further evidence that the *Picobirnaviridae* indeed have bacterial hosts.

The host range of virus species within each of the seven proposed *Picobirnaviridae* genera appeared to comprise either predominantly animal-associated picobirnaviruses (‘Alphapicobirnavirus’, ‘Betapicobirnavirus’, ‘Epsilonpicobirnavirus’, and ‘Zetapicobirnavirus’) or those identified in microbial or environmental samples (‘Gammapicobirnavirus’, ‘Deltapicobirnavirus’, and ‘Etapicobirnavirus’). However, the four genera comprising predominantly animal-associated viruses did not group together to the exclusion of the three genera comprising mainly microbe/environmentally sourced viruses. As such, this broad host-associated clustering may reflect the different microbial community compositions in terrestrial, aquatic, and engineered environments compared to those of mammals, birds, and invertebrates. Picobirnaviruses may infect different microbial organisms that play roles in food webs linking vertebrates, invertebrates, and their diets and habitats. This would facilitate the extensive ‘host-jumping’ observed within the animal-dominated genera. Hence, future metagenomic studies on the viromes of animal (particularly faecal) or environmental samples should also include community composition analyses of the microorganisms present. Determining if the microbial community composition of a sampled animal or environment is indeed driving the phylogenetic patterns of the *Picobirnaviridae* may elucidate the true host range of *Picobirnavirus* genera.

## Supplementary Material

veae033_Supp

## References

[R1] Adriaenssens E. M. et al. (2018) ‘Viromic Analysis of Wastewater Input to a River Catchment Reveals a Diverse Assemblage of RNA Viruses’, *mSystems*, 3: e00025–18. 29795788 10.1128/mSystems.00025-18 PMC5964442

[R2] Bell N. et al. (2020) ‘Molecular Detection and Characterization of *Picobirnavirus* in Environmental Water in Thailand’, *Clinical Laboratory*, 66: 855–8. 10.7754/Clin.Lab.2019.191013 32390390

[R3] Bonny P. et al. (2021) ‘Human and Animal RNA Virus Diversity Detected by Metagenomics in Cameroonian Clams’, *Frontiers in Microbiology*, 12: 770385. 10.3389/fmicb.2021.770385 PMC8669915 34917052

[R4] Boros A. et al. (2018) ‘Multiple Divergent Picobirnaviruses with Functional Prokaryotic Shine-Dalgarno Ribosome Binding Sites Present in Cloacal Sample of a Diarrheic Chicken’, *Virology*, 525: 62–72. 30245195 10.1016/j.virol.2018.09.008

[R5] Capella-Gutierrez S., Silla-Martinez J. M., and Gabaldon T. (2009) ‘trimAl: A Tool for Automated Alignment Trimming in Large-scale Phylogenetic Analyses’, *Bioinformatics*, 25: 1972–3. 19505945 10.1093/bioinformatics/btp348 PMC2712344

[R6] Chen Y.-M. et al. (2022) ‘RNA Viromes from Terrestrial Sites across China Expand Environmental Viral Diversity’, *Nature Microbiology*, 7: 1312–23. 10.1038/s41564-022-01180-2 35902778

[R7] Cole T. E. et al. (2000) ‘Detection of an RNA-dependent RNA Polymerase in Mitochondria from a Mitovirus-infected Isolate of the Dutch Elm Disease Fungus, *Ophiostoma Novo-ulmi*’, *Virology*, 268: 239–43. 10704332 10.1006/viro.1999.0097

[R8] Delmas B. et al. (2019) ‘ICTV Virus Taxonomy Profile’, *Journal of General Virology*, 100: 133–4. 30484763 10.1099/jgv.0.001186 PMC12662030

[R9] Duraisamy R. et al. (2018) ‘Detection of Novel RNA Viruses from Free-living Gorillas, Republic of the Congo: Genetic Diversity of Picobirnaviruses’, *Virus Genes*, 54: 256–71. 29476397 10.1007/s11262-018-1543-6 PMC7088520

[R10] Fong J. J. et al. (2012) ‘A Phylogenomic Approach to Vertebrate Phylogeny Supports a Turtle-archosaur Affinity and a Possible Paraphyletic Lissamphibia’, *PLoS One*, 7: e48990. 10.1371/journal.pone.0048990 PMC3492174 23145043

[R11] Fu L. et al. (2012) ‘CD-HIT: Accelerated for Clustering the Next-generation Sequencing Data’, *Bioinformatics*, 28: 3150–2. 23060610 10.1093/bioinformatics/bts565 PMC3516142

[R13] Gan T., and Wang D. (2023) ‘Picobirnaviruses Encode Proteins that are Functional Bacterial Lysins’, *Proceedings of the National Academy of Sciences*, 120: e2309647120. 10.1073/pnas.2309647120 PMC10500164 37669381

[R12] Ganesh B., Masachessi G., and Mladenova Z. (2014) ‘Animal Picobirnavirus’, *Virus Disease*, 25: 223–38. 25674589 10.1007/s13337-014-0207-y PMC4188182

[R15] Geoghegan J. L., Duchêne S., and Holmes E. C. (2017) ‘Comparative Analysis Estimates the Relative Frequencies of Co-divergence and Cross-species Transmission within Viral Families’, *PLoS Pathogens*, 13: e1006215. 10.1371/journal.ppat.1006215 PMC5319820 28178344

[R14] Geoghegan J. L. et al. (2021) ‘Virome Composition in Marine Fish Revealed by Meta-transcriptomics’, *Virus Evolution*, 7: veab005. 10.1093/ve/veab005 PMC7887440 33623709

[R16] Ghosh S., and Malik Y. S. (2021) ‘The True Host/s of Picobirnaviruses’, *Frontiers in Veterinary Science*, 7: 615293. 10.3389/fvets.2020.615293 PMC7855169 33553283

[R17] Giannitti F. et al. (2015) ‘Exploring the Virome of Diseased Horses’, *Journal of General Virology*, 96: 2721–33. 26044792 10.1099/vir.0.000199 PMC4635498

[R18] Gray S. M., and Banerjee N. (1999) ‘Mechanisms of Arthropod Transmission of Plant and Animal Viruses’, *Microbiology and Molecular Biology Reviews*, 63: 128–48. 10066833 10.1128/mmbr.63.1.128-148.1999 PMC98959

[R19] Guajardo-Leiva S. et al. (2020) ‘Metagenomic Insights into the Sewage RNA Virosphere of a Large City’, *Viruses*, 12: 1050. 10.3390/v12091050 PMC7551614 32967111

[R20] Hillman B. I., and Cai G. (2013) ‘The Family Narnaviridae: Simplest of RNA Viruses’, *Advances in Virus Research*, 86: 149–76. 23498906 10.1016/B978-0-12-394315-6.00006-4

[R21] Ho S. Y. W., Duchêne S., and Duchêne D. (2015) ‘Simulating and Detecting Autocorrelation of Molecular Evolutionary Rates among Lineages’, *Molecular Ecology Resources*, 15: 688–96. 25155426 10.1111/1755-0998.12320

[R22] Huang Y. et al. (2023) ‘A Global Dataset of Sequence, Diversity and Biosafety Recommendation of Arbovirus and Arthropod-specific Virus’, *Scientific Data*, 10: 305. 10.1038/s41597-023-02226-8 PMC10199016 37208388

[R23] Katoh K., and Standley D. M. (2013) ‘MAFFT Multiple Sequence Alignment Software Version 7: Improvements in Performance and Usability’, *Molecular Biology and Evolution*, 30: 772–80. 23329690 10.1093/molbev/mst010 PMC3603318

[R24] Kleymann A. et al. (2020) ‘Detection and Molecular Characterization of Picobirnaviruses (Pbvs) in the Mongoose: Identification of a Novel PBV Using an Alternative Genetic Code’, *Viruses*, 12: 99. 10.3390/v12010099 PMC7019992 31952167

[R25] Krishnamurthy S. R., and Wang D. (2018) ‘Extensive Conservation of Prokaryotic Ribosomal Binding Sites in Known and Novel Picobirnaviruses’, *Virology*, 516: 108–14. 29346073 10.1016/j.virol.2018.01.006

[R26] Le Lay C. et al. (2020) ‘Unmapped RNA Virus Diversity in Termites and Their Symbionts’, *Viruses*, 12: 1145. 10.3390/v12101145 PMC7650761 33050289

[R27] Lindblad-Toh K. et al. (2005) ‘Genome Sequence, Comparative Analysis and Haplotype Structure of the Domestic Dog’, *Nature*, 438: 803–19. 16341006 10.1038/nature04338

[R28] Mahar J. E. et al. (2020) ‘Comparative Analysis of RNA Virome Composition in Rabbits and Associated Ectoparasites’, *Journal of Virology*, 94: e02119–19. 32188733 10.1128/JVI.02119-19 PMC7269439

[R29] Malik Y. S. et al. (2014) ‘Epidemiology, Phylogeny, and Evolution of Emerging Enteric Picobirnaviruses of Animal Origin and Their Relationship to Human Strains’, *BioMed Research International*, 2014: 1–13. 10.1155/2014/780752 PMC4124650 25136620

[R30] Neri U. et al. (2022) ‘Expansion of the Global RNA Virome Reveals Diverse Clades of Bacteriophages’, *Cell*, 185: 4023–4037.e18. 36174579 10.1016/j.cell.2022.08.023

[R31] Nguyen L.-T. et al. (2015) ‘IQ-TREE: A Fast and Effective Stochastic Algorithm for Estimating Maximum-likelihood Phylogenies’, *Molecular Biology and Evolution*, 32: 268–74. 25371430 10.1093/molbev/msu300 PMC4271533

[R32] Paradis E., Schliep K., and Schwartz R. (2019) ‘Ape 5.0: An Environment for Modern Phylogenetics and Evolutionary Analyses in R’, *Bioinformatics*, 35: 526–8. 30016406 10.1093/bioinformatics/bty633

[R33] Prum R. O. et al. (2015) ‘A Comprehensive Phylogeny of Birds (Aves) Using Targeted Next-generation DNA Sequencing’, *Nature*, 526: 569–73. 26444237 10.1038/nature15697

[R34] Rambaut A. (2018), FigTree. <https://github.com/rambaut/figtree/> accessed 26 Mar 2019.

[R35] Sadiq S. et al. (2022) ‘Resolving Deep Evolutionary Relationships within the RNA Virus Phylum *Lenarviricota*’, *Virus Evolution*, 8: veac055. 10.1093/ve/veac055 PMC9252102 35795296

[R36] Santichaivekin S. et al. (2021) ‘eMPRess: A Systematic Cophylogeny Reconciliation Tool’, *Bioinformatics*, 37: 2481–2. 33216126 10.1093/bioinformatics/btaa978

[R37] Shen Y.-Y. et al. (2014) ‘The Updated Phylogenies of the *Phasianidae* Based on Combined Data of Nuclear and Mitochondrial DNA’, *PLoS One*, 9: e95786. 10.1371/journal.pone.0095786 PMC3991718 24748132

[R38] Shi M. et al. (2016) ‘Redefining the Invertebrate RNA Virosphere’, *Nature*, 540: 539–43. 27880757 10.1038/nature20167

[R39] Song S. et al. (2012) ‘Resolving Conflict in Eutherian Mammal Phylogeny Using Phylogenomics and the Multispecies Coalescent Model’, *Proceedings of the National Academy of Sciences*, 109: 14942–7. 10.1073/pnas.1211733109 PMC3443116 22930817

[R40] Urayama S., Takaki Y., and Nunoura T. (2016) ‘FLDS: A Comprehensive dsRNA Sequencing Method for Intracellular RNA Virus Surveillance’, *Microbes & Environments*, 31: 33–40. 26877136 10.1264/jsme2.ME15171 PMC4791113

[R41] Vainio E. J. et al. (2018) ‘ICTV Virus Taxonomy Profile: Partitiviridae’, *Journal of General Virology*, 99: 17–8. 29214972 10.1099/jgv.0.000985 PMC5882087

[R42] Wakuda M., Pongsuwanna Y., and Taniguchi K. (2005) ‘Complete Nucleotide Sequences of Two RNA Segments of Human Picobirnavirus’, *Journal of Virological Methods*, 126: 165–9. 15847933 10.1016/j.jviromet.2005.02.010

[R43] Wolf Y. I. et al. (2020) ‘Doubling of the Known Set of RNA Viruses by Metagenomic Analysis of an Aquatic Virome’, *Nature Microbiology*, 5: 1262–70. 10.1038/s41564-020-0755-4 PMC7508674 32690954

[R44] Woo P. C. Y. et al. (2019) ‘Novel Picobirnaviruses in Respiratory and Alimentary Tracts of Cattle and Monkeys with Large Intra- and Inter-host Diversity’, *Viruses*, 11: 574. 10.3390/v11060574 PMC6631280 31234565

[R45] Yinda C. K. et al. (2019) ‘Gut Virome Analysis of Cameroonians Reveals High Diversity of Enteric Viruses, Including Potential Interspecies Transmitted Viruses’, *mSphere*, 4: e00585–18. 30674646 10.1128/mSphere.00585-18 PMC6344602

[R0045a] ——— (2018) ‘Cameroonian fruit bats harbor divergent viruses, including rotavirus H, bastroviruses, and picobirnaviruses using an alternative genetic code’, *Virus Evolution*, 4: vey008. 10.1093/ve/vey008 PMC5888411 29644096

[R46] Yu G. et al. (2017) ‘ggtree : An R Package for Visualization and Annotation of Phylogenetic Trees with Their Covariates and Other Associated Data’, *Methods in Ecology and Evolution*, 8: 28–36.

[R47] Zhou Y., Wang S.-R., and Ma J.-Z. (2017) ‘Comprehensive Species Set Revealing the Phylogeny and Biogeography of Feliformia (Mammalia, Carnivora) Based on Mitochondrial DNA’, *PLoS One*, 12: e0174902. 10.1371/journal.pone.0174902 PMC5373635 28358848

[R48] Zurano J. P. et al. (2019) ‘Cetartiodactyla: Updating a Time-calibrated Molecular Phylogeny’, *Molecular Phylogenetics & Evolution*, 133: 256–62. 30562611 10.1016/j.ympev.2018.12.015

