# Supplementary material for: Genomic and phylogenetic features of the Picobirnaviridae suggest microbial rather than animal hosts
Source: Virus Evol. 2024 Apr 22;10(1):veae033. doi: 10.1093/ve/veae033 (PMC11096803; doi:10.1093/ve/veae033)
Supplement: veae033_Supp [file veae033_supp.zip › SUPPLEMENTARY INFORMATION.docx]

**SUPPLEMENTARY INFORMATION**

**Supplementary Table 1.** *Picobirnaviridae* sequences able to be classified into one of seven proposed genera, including assigned host organism or sampling environment.

**Supplementary Figure 1.** Maximum likelihood phylogenetic tree of the RdRp of proposed genus *Alphapicobirnavirus*. Tip labels are coloured to represent assigned host/sampling environment. Sequences utilising an alternative genetic code are marked by an asterisk (*) followed by the genetic code and corresponding translation table number. Branch lengths represent the number of amino acid substitutions per site, indicated by the scale bar. The tree is midpoint rooted for clarity only.

**Supplementary Figure 2.** Maximum likelihood phylogenetic tree of the RdRp of proposed genus *Betapicobirnavirus*. Tip labels are coloured to represent assigned host/sampling environment. Sequences utilising an alternative genetic code are marked by an asterisk (*) followed by the genetic code and corresponding translation table number. Branch lengths represent the number of amino acid substitutions per site, indicated by the scale bar. The tree is midpoint rooted for clarity only.

**Supplementary Figure 3.** Maximum likelihood phylogenetic tree of the RdRp of proposed genus *Gammapicobirnavirus*. Tip labels are coloured to represent assigned host/sampling environment. Animal hosts are categorised broadly as ‘mammalian’, ‘avian’, and ‘invertebrate’, and non-animal associated sequences are grouped into ‘environmental’ or ‘microbial’ samples, both further classed into ‘terrestrial’, ‘aquatic’, and ‘engineered’ sampling sources. Sequences utilising an alternative genetic code are marked by an asterisk (*) followed by the genetic code and corresponding translation table number. Branch lengths represent the number of amino acid substitutions per site, indicated by the scale bar. The tree is midpoint rooted for clarity only.

**Supplementary Figure 4.** Maximum likelihood phylogenetic tree of the RdRp of proposed genus *Deltapicobirnavirus*. Tip labels are coloured to represent sampling environment. Sequences are grouped into ‘environmental’ or ‘microbial’ samples, both further classed into ‘terrestrial’ and ‘aquatic’. Branch lengths represent the number of amino acid substitutions per site, indicated by the scale bar. The tree is midpoint rooted for clarity only.

**Supplementary Figure 5.** Maximum likelihood phylogenetic tree of the RdRp of proposed genus *Epsilonpicobirnavirus* Tip labels are coloured to represent assigned host/sampling environment. Branch lengths represent the number of amino acid substitutions per site, indicated by the scale bar. The tree is midpoint rooted for clarity only.

**Supplementary Figure 6.** Maximum likelihood phylogenetic tree of the RdRp of proposed genus *Zetapicobirnavirus*. Tip labels are coloured to represent assigned host. Sequences utilising an alternative genetic code are marked by an asterisk (*) followed by the genetic code and corresponding translation table number. Branch lengths represent the number of amino acid substitutions per site, indicated by the scale bar. The tree is midpoint rooted for clarity only.

**Supplementary Figure 7.** Maximum likelihood phylogenetic tree of the RdRp of proposed genus *Etapicobirnavirus*. Tip labels are coloured to represent assigned host animal. Animal hosts are categorised broadly as ‘mammalian’, ‘avian’, “fish”, “reptile”, and ‘invertebrate’, and non-animal associated sequences are grouped into ‘environmental’ or ‘microbial’ samples, both further classed into ‘terrestrial’, ‘aquatic’, and ‘engineered’ sampling sources. Branch lengths represent the number of amino acid substitutions per site, indicated by the scale bar. The tree is midpoint rooted for clarity only.

**Supplementary Figure 8.** Maximum likelihood trees of the RdRp of animal-associated *Picobirnaviridae* RdRp with tip labels coloured to represent assigned host/sampling environment. *Partitiviridae* RdRp sequences (n=40) were used as an outgroup and are shown in black. Branch lengths represent the number of amino acid substitutions per site, indicated by the scale bar.

**Supplementary Figure 9.** Maximum likelihood trees of the RdRp of *Picobirnaviridae* RdRp with tip labels coloured to represent assigned host animal. *Partitiviridae* RdRp sequences (n=40) were used as an outgroup and are shown in black. Branch lengths represent the number of amino acid substitutions per site, indicated by the scale bar.

**Supplementary Figure 10.** Maximum likelihood trees of the RdRp of animal-associated *Picobirnaviridae* RdRp with tip labels coloured to represent assigned host/sampling environment. *Partitiviridae* RdRp sequences (n=40) were used as an outgroup and are shown in black. Animal hosts are categorised broadly as ‘mammalian’, ‘avian’, “fish”, “reptile”, and ‘invertebrate’. Branch lengths represent the number of amino acid substitutions per site, indicated by the scale bar.

**Supplementary Figure 11.** Maximum likelihood trees of the RdRp of *Picobirnaviridae* RdRp with tip labels coloured to represent assigned host/sampling environment. *Partitiviridae* RdRp sequences (n=40) were used as an outgroup and are shown in black. Animal hosts are categorised broadly as ‘mammalian’, ‘avian’, “fish”, “reptile”, and ‘invertebrate’, and non-animal associated sequences are grouped into ‘environmental’ or ‘microbial’ samples, both further classed into ‘terrestrial’, ‘aquatic’, and ‘engineered’ sampling sources. Branch lengths represent the number of amino acid substitutions per site, indicated by the scale bar.

**Supplementary Figure 12.** Distribution of assigned hosts and sampling sourced for each proposed *Picobirnaviridae* genus, represented as relative proportions of the total number of contigs comprising each proposed genus. For each proposed genus, two bar charts are shown. The bar chart on the left in each pair (labelled “Animal”) is coloured by animal hosts, with environmental and microbially-sourced sequences coloured in black, as per the animal-focused colour legend (top). In the bar chart on the right in each pair (“Env/microbial”), animal hosts are categorised broadly as ‘mammalian’, ‘avian’, “fish”, “reptile”, and ‘invertebrate’, and non-animal associated sequences are grouped into ‘environmental’ or ‘microbial’ samples, both further classed into ‘terrestrial’, ‘aquatic’, and ‘engineered’ sampling sources, as per the environmental/microbial-focused colour legend (bottom).

**Supplementary Figure 13.** Cladogram of host animals. Topology is based on current literature on the evolutionary history of vertebrates and invertebrates. The tree was manually written in Newick format. A scale bar is not included as branch lengths do not represent any actual changes in genetic sequence.
